# Troubleshooting intrathecal pumps in pain management: A clinical review

**DOI:** 10.1016/j.inpm.2026.100747

**Published:** 2026-03-04

**Authors:** Natalia Wojnowski, Salim M. Hayek, Shanee Abouzaglo, Kelly Li, R. Jason Yong, David Hao

**Affiliations:** a Department of Physical Medicine and Rehabilitation, Harvard Medical School/Spaulding Rehabilitation Hospital, Boston, MA, USA; b University Hospitals Cleveland Medical Center, Case Western Reserve University School of Medicine, Cleveland, OH, USA; c Harvard Medical School, Boston, MA, USA; d Department of Anesthesiology, Mass General Brigham, Boston, MA, USA

**Keywords:** Intrathecal drug delivery, Device troubleshooting, Chronic pain

## Abstract

Intrathecal drug delivery systems (IDDS), commonly known as pain pumps, have become an increasingly more prevalent method for management of refractory chronic pain, both non-cancer and cancer-related pain. IDDS are generally regarded as safe and reliable, however there is potential for complications leading to clinically significant consequences, such as accidental overdose, withdrawal and neurologic injury. In cases of suspected device malfunction, it is important to have an organized approach to troubleshooting. IDSS are also utilized for intrathecal baclofen therapy, and several prior reviews have focused on algorithms for management of ITB-related pump problems. While there is overlap with existing literature on intrathecal baclofen therapy, the clinical presentation and work-up varies when utilizing opioids and local anesthetics. Therefore, we propose a stepwise framework for troubleshooting intrathecal pump therapy in the setting of non-baclofen medications.

## 1 Introduction

**Fig. 1:**
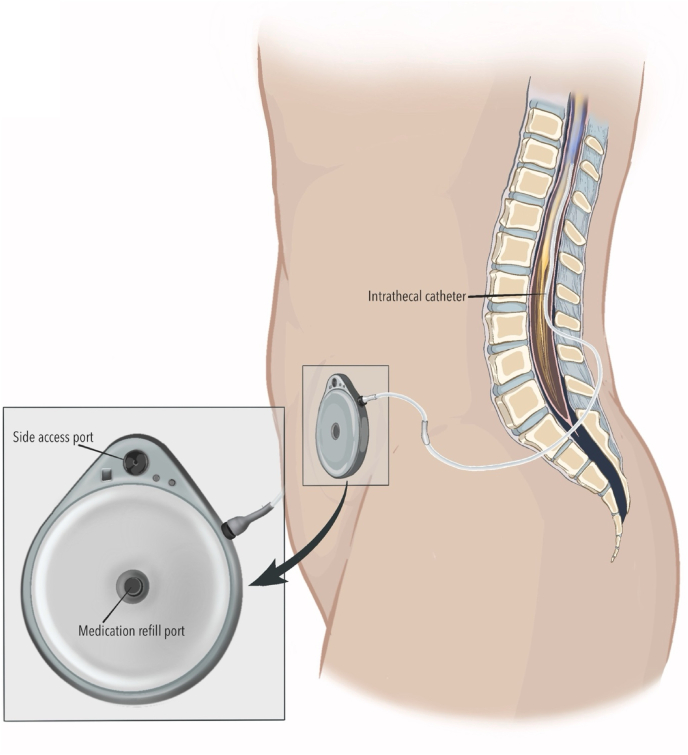
Intrathecal drug delivery system in situ The pump is positioned in the lower abdominal quadrant and connected to a catheter inserted into the intrathecal space.

Implantable devices that deliver analgesic medications directly into the cerebrospinal fluid (CSF), known as intrathecal drug delivery systems (IDDS), provide targeted pain control for patients with severe, refractory chronic non-cancer and cancer-related pain [1,2] (Fig. 1). With ongoing technological advancements, IDDS are generally safe and reliable [3]. However, complications can occur from device-related issues or human-factor contributors, which can have clinically significant consequences [4]. Disruptions in drug delivery may arise from damaged or occluded catheters, pump motor stalls, valve or septum malfunction, and errors during IDDS programming or refilling, among other causes. Malfunctions or errors can lead to drug underdelivery, resulting in loss of analgesic efficacy or withdrawal, or rarely to drug overdelivery, risking sedation and medication overdose [5].

Therefore, it is crucial for clinicians to undertake a systematic, evidence-based approach to troubleshooting that integrates clinical evaluation, device interrogation, imaging, and testing to identify the source of IDDS dysfunction and guide prompt intervention to prevent serious complications. Currently, there are two manufacturers of IDDS, Medtronic and Flowonix. Device-specific manufacturer recommendations are available and can be referenced for additional information.

Prior reviews have largely focused on intrathecal baclofen (ITB) therapy and have proposed algorithmic approaches to the evaluation and management of ITB pump-related problems [6,7]. The delivery systems themselves are mechanically identical across indications; however, the clinical presentation and diagnostic work-up may differ when agents like opioids and local anesthetics are used. This manuscript therefore focuses on troubleshooting intrathecal pump therapy in the setting of non-baclofen medications while acknowledging overlap with existing ITB-focused frameworks.

### 1.1 Overview

Malfunction of IDDS systems can occur for a variety of reasons. The American Society of Pain and Neuroscience (ASPN) has compiled a list of causes for IDDS pump failure in their best practices and guidelines for interventional management of cancer pain. These include change in performance or failure of the catheter (micro-fracture, pinhole leak, disconnection, breakage, migration, partial occlusion, tip fibrosis, inflammatory mass), unexpected battery depletion, component or motor failure and catheter access port failure [8]. In an analysis of adverse events and complications related to IDDS reported in the FDA MAUDE database, the authors categorized adverse events as catheter-related, pump-related, biologic and medication-related. Among the adverse events that contributed to IDDS malfunction, there were 249 catheter-related complications, 357 pump-related complications with motor stall making up one-third of all pump complications and 19 cases of granuloma formation [4].

## 2 Clinical assessment and pump interrogation

When evaluating a patient with a suspected IDDS malfunction, a prompt and focused clinical assessment should be performed to narrow the differential diagnosis and guide diagnostic evaluations and interventions [6,9]. An important early step is confirming that the patient has an IDDS, identifying the device model, and verifying the medication dosages and concentrations being infused.

The history should then clarify presenting symptoms such as changes in pain level or sedation, their onset and severity, whether they correlate with recent programming adjustments or refills, and any recent medication changes, procedures, or imaging that could contribute to the presentation. Patient history is also critical because prior episodes of similar symptoms may offer clues to the underlying cause and help identify an inciting event. It is also essential to consider alternative explanations for worsening pain, including disease progression or new injury.

The interrogation report provides critical information, including whether the pump has stalled, medications and concentrations in the reservoir, programmed dosing and delivery parameters, implant details, reservoir calculated medication volume, recorded errors or alarms, and the recommended pump replacement date (elective replacement indicator). The device interrogation should be performed using the clinician programmer that communicates with the pump.

Finally, the pump's event log should also be reviewed for alarm events, battery status indicators (low battery reset (LBR), elective replacement indicator (ERI), end of service (EOS)), motor function, patient-controlled intrathecal analgesia (PCIA) activations, and programming changes [10].

### 2.1 Audible alarms

Implantable intrathecal pumps are equipped with audible alarms designed to alert patients and clinicians to conditions that require attention. The pattern, frequency, and urgency of the alarm depend on the manufacturer and device model. When an audible alarm is reported or detected, immediate pump interrogation is essential to review the event log and assess current delivery status.

Flowonix Prometra pumps generate two primary audible alarm patterns. The Low Reservoir Alarm signals that the medication volume has fallen below a preset threshold and is characterized by two short (approximately 0.25-s) beeps every 30 min. This alarm persists until it is silenced via telemetry with the Prometra Pump Programmer or until the reservoir is refilled and a new volume is programmed. The Critical Error Alarm indicates that the pump has stopped delivering medication. It is characterized by three long (approximately 0.5-s) beeps every 30 min and may occur due to several conditions, including pump battery depletion or other system faults [11].

Similarly, Medtronic SynchroMed pumps have two audible alarms that indicate to the patient that there is a need for medical evaluation. The Critical Alarm is a 3-s dual-tone alarm and indicates imminent cessation of therapy. This may occur due to the pump being empty (estimated reservoir volume is 0.0 ml), end of service requiring replacement, detected motor stall or prolonged shutdown (>48 h). The Non-Critical Alarm is a single-tone indicating that the pump is nearing replacement time or the reservoir volume is low. It is characterized by a single beep that repeats about every hour. The alarm is activated when the estimated reservoir volume reaches the empty volume alarm threshold. The manufacturer default setting is 2.0 ml, but the low volume threshold at which the alarm is activated can be adjusted by the clinician. Additionally, the elective replacement indicator triggers a Non-Critical Alarm 90 days prior to need for replacement. Audible alarms can be silenced, but the alarm will be recorded with a time-stamp in the Event Log and silencing the alarm does not resolve the condition causing the alarm. Additionally, it is important to note Non-Critical Alarms will not sound if a Critical Alarm is activated as Critical Alarms take precedence over Non-Critical Alarms [12].

## 3 Clinical presentations

It is particularly important to evaluate for signs or symptoms of drug withdrawal or overdose such as diaphoresis, agitation and/or anxiety, tachycardia and/or hypertension, and diarrhea; or conversely overdose such as sedation, somnolence, respiratory depression, bradycardia, or hypotension. Interpreting these findings is best guided by a clear understanding of the medications and concentrations in the IDDS, as the central goal in these scenarios is determining the extent to which the pump is contributing to the patient's presentation.

Although only morphine, ziconotide, and baclofen are FDA-approved monotherapy agents for intrathecal use, real-world practice differs substantially. In a large retrospective analysis of 32,784 patients and 49,917 prescriptions over one year, only 3952 prescriptions met FDA-approved indications for both agent and concentration, representing 7.9 percent of all prescriptions. These findings underscore the importance of understanding the specific agents and concentrations present in an individual patient's pump to guide clinical assessment and decision-making [13].

### 3.1 Withdrawal presentation

To specifically characterize intrathecal opioid withdrawal patterns, Jackson et al. followed three patients after controlled, abrupt IDDS cessation. Patients were monitored using the Clinical Opiate Withdrawal Scale and managed with supportive care. The authors described a constellation of withdrawal features including hypertension, hyperalgesia, restlessness, myalgias, yawning, dysphoria, taste/smell aversion, and diuresis. In contrast, several classic symptoms of opioid withdrawal, such as nausea, vomiting, diarrhea, piloerection, diaphoresis, chills, mydriasis, and myoclonus, were not observed, suggesting the possibility of a distinct “spinal withdrawal.” [14].

In a 2004 study, Taha et al. observed that 24% (21/88) of patients with implanted intrathecal SynchroMed pumps reported symptoms of medication withdrawal 1-7 days before their scheduled pump refills despite residual volumes measuring, on average, 2.7 mL (2.1-3.8 mL) – above the manufacturer's 2 mL (mL) alarm threshold [15]. Symptoms were reported within 1-18 months post-implantation and symptoms resolved after refill without dose changes and did not recur after increasing the alarm to 4 mL. No predisposing factors (daily flow volumes, medication dose, concentration and combinations, oral medications, disease states) were identified. Although the specific pump model was not explicitly stated, implants occurred between 1996 and 2003, suggesting use of earlier-generation SynchroMed pumps. The authors proposed that “erratic flow” at residual volumes between 2 and 4 mL may result in inconsistent drug delivery, leading to functional under-delivery and withdrawal symptoms despite apparently adequate residual reservoir volume [15].

There are a few additional case reports describing withdrawal from pump malfunctions. In one case, a patient with a Medtronic SynchroMed EL programmable infusion pump presented to the emergency department with pain, jitteriness, and fatigue 32 h after pump refill. The patient was being treated with 13 mg morphine and 2.6 mg bupivacaine per day. On evaluation, a needle was used to aspirate the pump contents, and 30 mL of clear fluid was withdrawn despite the reservoir capacity being 18 mL. The fluid was sent for testing and found to be positive for glucose, suggesting it was cerebrospinal fluid. During intraoperative pump replacement, the connector between the pump and intrathecal catheter was found to be broken, resulting in pooling of medication in a pocket surrounding the pump [16].

### 3.2 Overdose presentation

Maino et al. described two cases of fentanyl overdose related to IDDS malfunction in the setting of high concentrations of fentanyl in the pump (3 and 6 mg/mL). In the first case, the patient presented to her clinician with increased sleepiness and significant pain reduction over several weeks. When the reservoir was emptied, the residual volume was less than expected, suggesting that a higher dose was being delivered than programmed. The SynchroMed II pump was explanted and sent to the manufacturer for analysis, which demonstrated overinfusion likely secondary to partial tubing occlusion due to drug precipitant deposition in the inner tubing resulting in continuous rather than intermittent infusion.

In the second case, a patient underwent a routine fentanyl refill of her SynchroMed II pump, and experienced dizziness approximately 5 h later. She was subsequently found unresponsive by her husband 10 h after refill and required evaluation in the emergency department. Overinfusion was again suspected, and the pump was removed and sent to the manufacturer for analysis, which revealed motor stall likely secondary to corrosion from the lipophilic (fentanyl) medication leak through the internal silicon tubing. Examination of the inner tubing demonstrated wear and rigidity, leading the authors to postulate that tubing degradation may have permitted drug leakage into the pump mechanism, resulting in corrosion [17].

The same group performed a prospective observational study of 221 refill procedures in 19 patients with MedStream (no longer marketed) programmable infusion systems, comparing injected drug volume with the volume measured within the IDDS reservoir and correlating volume discrepancies with clinical signs of overdose. Six overdose events occurred in four patients, all following fentanyl refills, with symptom onset 5 to 90 min after the procedure. The most common signs were somnolence and respiratory depression (including desaturation), with additional manifestations of pinpoint pupils, tremor, rigidity, nausea, and vomiting [18].

### 3.3 Laboratory assessment

As part of an initial evaluation, serum studies may be useful to assess for infection or toxic-metabolic derangements that may mimic or exacerbate symptoms attributable to IDDS malfunction. Recommended laboratory studies include complete blood count, complete metabolic panel, and coagulation studies [6]. Leukocytosis may suggest an infectious or inflammatory process, while anemia may present with non-specific weakness or fatigue. Electrolyte or other metabolic derangements can manifest as neuromuscular symptoms; evaluating kidney and liver function is important because dysfunction may impair metabolism and clearance of medications. Additionally, it may be appropriate to check creatine phosphokinase (CK) levels, as rising CK may be a side effect of certain opioids and non-opioid analgesics (ie. ziconotide) and can result in myopathy and rhabdomyolysis [19]. These tests may identify alternative or concurrence processes that guide a differential diagnosis, but laboratory studies do not “rule in” or “rule out” specific IDDS-related issues.

## 4 External environmental factors affecting pump function

It is important to ask about recent MRIs, shock wave lithotripsy, radiation therapy [20] SCUBA diving [21] and hyperbaric oxygen therapy, as these external exposures can damage the pump or cause temporary changes in drug infusion [6].

### 4.1 Magnetic resonance imaging

As IDDS are MRI “conditional”, it is important to confirm adherence to MRI protocols based on IDDS manufacturer guidelines [22].

Medtronic SynchroMed II and III pumps are deemed safe for MRI exams of the entire body if the following conditions are met: 1.5-T and 3-T horizontal cylindrical system for hydrogen imaging, any type radiofrequency coil, maximum spatial gradient of 19T/m, max gradient slew of 200T/m/s or less per axis, maximum RF field intensity of First Level Controlled Operating Mode and active scan time of less than 30 min. There is an increased risk of tissue heating adjacent to the implanted pump for scans of the torso exceeding 30 min. Scans should be stopped if a patient indicates discomfort in the area. There are currently no established guidelines for pump performance in the use of open-sided or standing MRIs [23].

For Flowonix Prometra pumps, patients undergoing MRI must have the pump reservoir completely emptied of medication, and the pump should be programmed to a flow rate of 0.0 mg/day prior to MRI exposure and throughout duration of the procedure. Additionally, the following MRI specifications are required: magnetic field of 1.5T, maximum spatial gradient of 410 Gauss/cm, and maximum whole body average specific absorption rate of 2W/kg for 20 min of scanning. Given the risk of tissue heating adjacent to the implant, MRI scans should be stopped if a patient experiences uncomfortable warmth near the pump. Upon completion of MRI, the pump should be interrogated with the programmer and pump errors cleared. Pump flow rate should be confirmed as 0.0 mg/day, followed by pump aspiration. If a volume >1 mL is obtained, this may be indicative of the pump valves being open causing direct access to the CSF in which case the pump should not be refilled but rather explanted and replaced. If the pump is operating appropriately, the clinician may proceed with pump refill and confirm the correct drug is programmed at the desired flow rate [11].

A 1991 study assessed the effects of MRI on IDDS by testing 6 identical Medtronic SynchroMed pumps taped to the body of a volunteer. They found that with 1.5T MRI, there was no structural damage to the pump, pump memory was not affected, however the pump rotor stalled while the device was in the magnetic field, and drug delivery resumed after the pump was removed from the magnetic field [24]. Kosturakis and Gebhardt reported a case of a patient who had a SynchroMed II intrathecal pump and underwent 11 MRIs, after which the patient noticed the pump alarming and experienced symptoms of withdrawal for which he presented to an emergency department. The pump was interrogated demonstrating a memory failure, but was able to be reprogrammed. The patient underwent an additional 7 MRIs over a 2.5 year period after which he experienced a second episode of withdrawal the day after an MRI and the pump was found to be malfunctioning and ultimately explanted and replaced [25]. This study emphasized the importance of pump interrogation every time a patient undergoes MRI given the possible complications from motor stall and memory errors.

## 5 Assessment of system integrity and flow

If the pump interrogation and aforementioned processes do not yield a “diagnosis”, the next step in troubleshooting an IDDS involves assessing the integrity and flow of the system by performing reservoir volume reconciliation and catheter assessment/access port aspiration.

### 5.1 Reservoir volume reconciliation

**Fig. 2:**
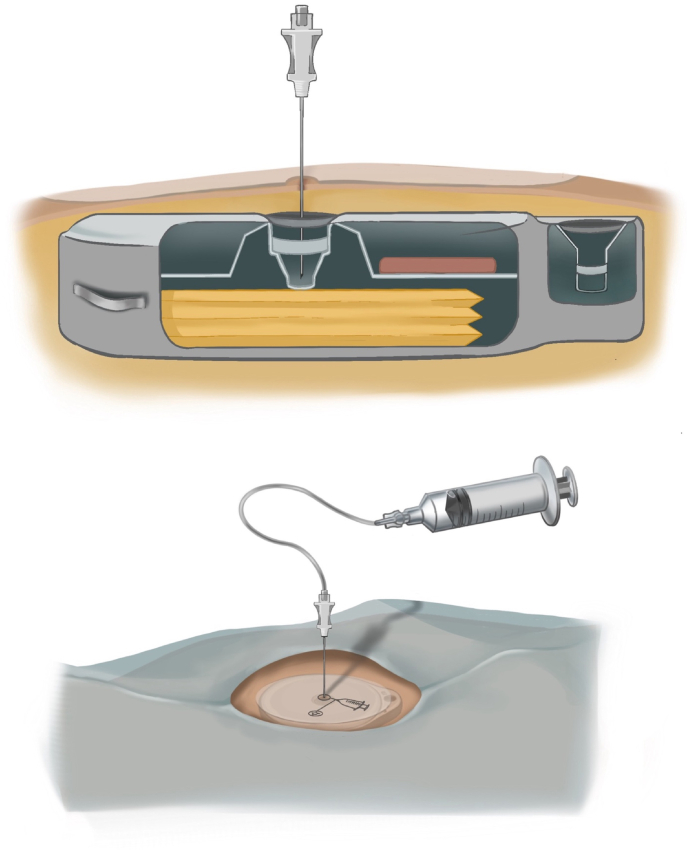
Reservoir volume reconciliation and refill technique with template After skin preparation and sterile draping, a template is used to identify the reservoir fill port. The needle is inserted perpendicular to the skin through the septum.

Volume discrepancy in an IDDS occurs when there is a mismatch between the expected volume the interrogation report predicts and the volume actually obtained when the reservoir is aspirated. Confirming a discrepancy therefore requires aspirating the volume to directly measure the remaining reservoir volume (Fig. 2). It is important to note that the low reservoir alarm is based on programmed settings rather than an actual measurement of the reservoir volume in both the Medtronic and Flowonix pumps, and therefore reflects a calculated value [10].

An excess residual volume suggests possible drug underdelivery due to catheter occlusion, pump stall, or rotor malfunction. In contrast, a lower-than-expected volume suggests possible drug leakage, premature emptying, or pocket fill.

In an analysis of adverse events related to intrathecal drug-delivery systems reported in the FDA MAUDE database, the authors identified 50 cases (4.9%) of incidental high residual volume during refills. Only twelve of the 50 patients reported uncontrolled pain or withdrawal which suggests that only a minority of patients may be symptomatic even in the setting of excess volume. While the exact causes of the excess residual volume were not specifically identified in this study, the patients were treated with catheter replacement, reduction in refill volume, device explantation or replacement of the system [4]. This study additionally identified 51 adverse events of catheter occlusion in which just over half of the patients were symptomatic. This same study also reported on the number of pump stall events. Of the 125 pump stall cases, 40 patients presented with worsening pain or withdrawal, 30 with device alarms and 28 cases were incidentally identified while the device was interrogated [4]. Motor stalls have been described in SynchroMed II pumps delivering predominantly lipophilic intrathecal agents, with no further reports published likely related to device improvements introduced by the manufacturer in 2017 [26]. A recent report of IDDS complications cited three valve-gated pump failures out of 14 implanted pumps [27].

Lower than expected volumes can be potentially dangerous as the patient may be receiving higher amounts of the drug than intended. One possible mechanism for lower-than-expected reservoir volume is drug leakage. Perruchoud et al. described a case of medication leakage through the septum due to damage from needle insertion resulting in lower than expected reservoir volume and symptoms of medication overdose [28].

A pocket fill, which occurs when the drug is inadvertently injected into the subcutaneous space, is particularly dangerous as it can result in systemic drug absorption leading to acute overdose. In the analysis of IDDS adverse events reported in the FDA MAUDE database, there were seven documented pocket fill events, all of which required emergency department visits or hospitalization for monitoring, underscoring the potential severity of systemic drug absorption from subcutaneous injection [4]. In a case report by Coyne et al., the authors described a case where a trained clinician was attempting to refill an intrathecal pump, however the patient moved resulting in the needle dislodging and a two-month supply of hydromorphone being injected into a subcutaneous pocket. If a pocket fill is suspected, the pump reservoir should be accessed to determine if any fluid can be aspirated. The authors outlined a series of steps to take when pocket fill is suspected, including: 1) recognizing the situation and notifying the appropriate personnel; 2) checking vital signs, assessing the level of sedation and pain, monitoring respiratory rate and oxygen saturation, and obtaining IV access; 3) preparing for a potential ICU transfer; 4) querying the pump, turning it off, and aspirating any opioid present to reduce further medication infusion; and 5) notifying risk management [17]. However, pocket fills are not always immediately recognized and should remain on the differential diagnosis.

In another study looking at volume discrepancy, the authors reviewed 221 refill procedures in 19 patients concluding that a volume discrepancy of >1 mL in 20 mL IDDSs and >2 mL in 40 mL IDDSs after refill should be considered potentially consequential. These volume thresholds were determined by the amounts at which patients experienced symptoms of overdose, 1.15-4.5 mL in 20 mL IDDS and between 2.09 and 4.88 mL in 40 mL IDDS. In this context, a negative volume discrepancy, where the measured reservoir volume is less than the expected volume by these margins, may be interpreted as apparent over-infusion. Given this increased risk of overdose, they recommended hospitalization and monitoring to prevent potentially dangerous overdose symptoms from pocket fill [18]. Clinicians should document volume discrepancies to inform future troubleshooting or surgical planning.

### 5.2 Catheter access port aspiration

**Fig. 3:**
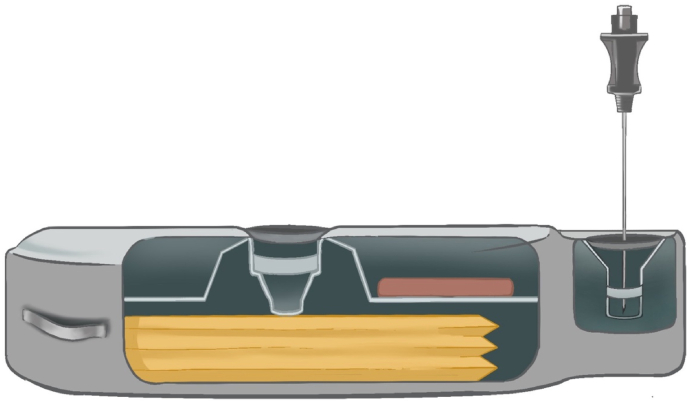
Needle access of catheter access port Schematic demonstrating needle insertion into catheter access port for diagnostic aspiration or contrast injection.

**Fig. 4:**
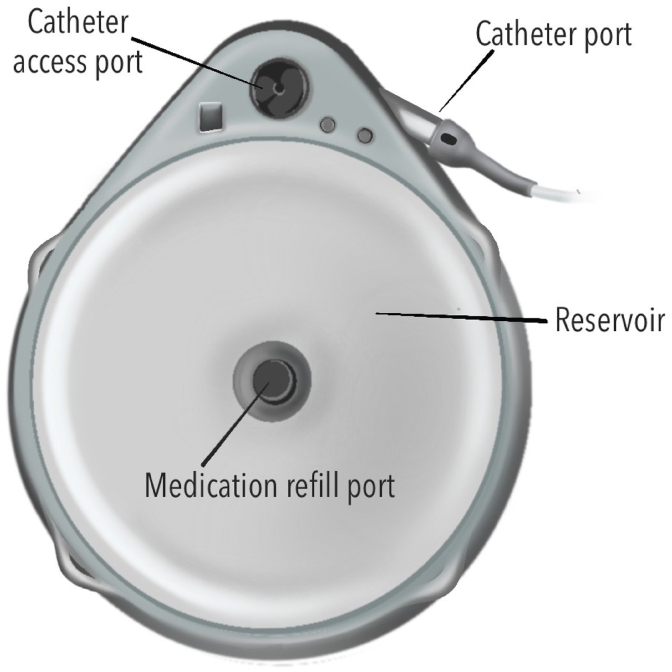
En face view of an intrathecal drug delivery system pump. The medication refill port is located at the center of the pump and has a septum that can be traversed with a needle to access the reservoir where the medication is stored. The catheter access port connects directly to the catheter.

A catheter access port (CAP) aspiration can be performed to assess catheter patency and to evaluate suspected catheter obstruction or disconnection (Fig. 3). In pump models that contain a CAP, aspiration should be attempted under strict aseptic technique and is preferably performed under fluoroscopy (Fig. 4). Fluoroscopy facilitates accurate localization of the CAP funnel, which may not be reliably identifiable in all cases, and reduces the risk of inadvertent damage to pump components [29]. After the port is localized, gentle aspiration should be performed; easy withdrawal of 2-3 mL of clear CSF is suggestive of catheter patency and communication with the intrathecal space [6]. In contrast, the inability to aspirate CSF, therefore, suggests possible obstruction, disconnection of the catheter, kinking or occlusion of the catheter tip.

The CAP can often be localized with a manufacturer template; however, in challenging cases, imaging guidance can be used to ensure accurate needle placement. Shankar described the use of ultrasound to access a refill port obscured by a seroma, allowing direct visualization of the seroma, reservoir, and refill point to facilitate safe and accurate needle placement [30].

The clinical importance of careful catheter assessment is underscored by adverse event data. In an analysis of IDDS-related events reported to the FDA MAUDE database, 82 cases involved catheter damage, including severing, nicking, fracture, or breakage. An additional 53 reports described catheter kinking and 51 reported catheter occlusion, together accounting for a substantial portion of adverse effects reported in the study [4].

**Fig. 5:**
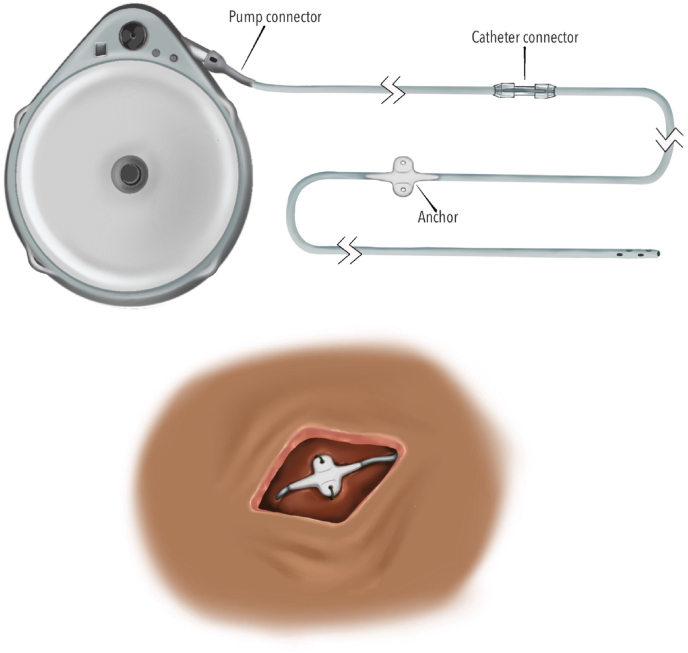
Components of the intrathecal drug delivery system The system consists of a pump and catheter. The pump connector links the pump to the catheter or extension catheter. The anchor is sutured to fascia to secure catheter positioning. Catheter disruptions may occur at any point along this pathway, including the pump connector, catheter connections, anchor site, and fascial entry point.

Catheter non-patency does not appear to have a single predominant etiology (Fig. 5). In a study looking at catheter patency in Synchromed II pump models, Skalsky et al. screened catheters beginning at the second refill appointment, with repeat assessment by a second clinician to reduce false-positive findings. Among 15 non-patent catheters, no identifiable cause was found in 7 cases, while 6 were broken, 1 was occluded and 1 was taught and kinked, leading the authors to conclude that non-patency arises from heterogenous mechanisms [31].

Finally, catheter-related complications appear comparable across different system designs. Follett et al. investigated complication rates in one-piece versus two-piece catheters systems and found similar incidences between designs. Among 209 participants, seven catheter-related complications were reported, including three cases of leakage, cuts or breaks, two cases of catheter dislodgement or migration, one catheter-to-pump connector issue and one catheter or pump disconnection [32].

## 6 Imaging studies

Imaging for troubleshooting an IDDS should be performed in a stepwise fashion [7,33]. Dvorak et al. proposed a troubleshooting flow sheet for intrathecal baclofen pump-catheter systems which provides a structured framework for imaging after completion of a clinical examination and pump interrogation. In this algorithm, imaging progresses from plain radiographs to fluoroscopic assessment with radiopaque dye instilled through the CAP, followed by computed tomography, with scintigraphy reserved as the final step [34]. Miracle et al. published a similar tiered approach for imaging evaluation of IDDS used for baclofen, likewise recommending initial radiographs and fluoroscopy, followed by CT and nuclear scintigraphy [33].

### 6.1 Radiographic and fluoroscopic evaluation including catheter access port myelography

Plain radiographs, including anteroposterior and lateral views of the abdomen and thoracolumbosacral spine, are the recommended first-line imaging modality to evaluate for catheter and pump integrity and positioning. These are a low-cost, noninvasive, and readily available imaging modality [34].

Discontinuities in the catheter may suggest disconnection, whereas sharp angles may represent kinking. In the troubleshooting algorithm proposed by Dvorak et al., plain radiographs alone identified approximately 35% of all catheter malfunctions in patients with intrathecal baclofen therapy [34]. It should be noted that some catheters are made of radiolucent material and may not be visible on roentgenograms.

When standard radiographs are nondiagnostic, serial radiographs or fluoroscopy may be used to assess pump migration or rotation and are particularly helpful when pump anchoring issues are suspected. Fluoroscopy evaluation commonly incorporates a catheter-dye study, often referred to as a “pump-o-gram”, to further assess catheter integrity. These studies are typically preceded by aspiration of the CAP to reduce the risk of unintended intrathecal drug bolus during contrast injection.

**Fig. 6:**
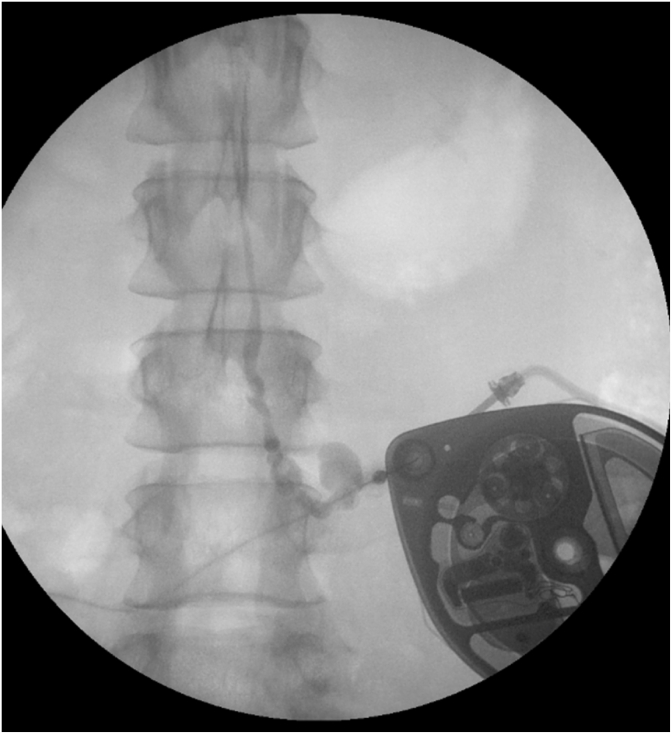
Catheter access port (CAP) contrast study Injection of iohexol through the CAP revealed contrast leak from the catheter in the lumbar region prior to entering the spinal canal. The catheter appears ectatic distal to the leak.

During a catheter dye study, the CAP is accessed with an appropriately sized non-coring needle under sterile conditions and aspiration is first attempted. Successful withdrawal of cerebrospinal fluid in a volume exceeding the expected catheter volume suggests catheter patency and communication with the intrathecal space, after which nonionic, preservative-free contrast may be injected under fluoroscopic guidance. Abnormal contrast patterns may identify catheter disconnection from the pump, large perforations or leaks and catheter-tip dislodgement or migration that are not apparent on plain radiographs [35] (Fig. 6). After the study, the contrast should be cleared from the system by programming a single bolus equivalent to the catheter tubing volume containing contrast.

If CSF cannot be aspirated, proceeding with contrast injection requires careful consideration, as a bolus injection may displace residual drug within the catheter into the intrathecal space. The decision to inject contrast should therefore incorporate the patient's clinical status, the expected volume and type of medication within the catheter based on calculated catheter dead space (i.e., the potential injected dose), and prior response or tolerability to pump medications. In some cases, hospital admission for monitoring and supportive care may be necessary [5,33,35].

The diagnostic limitations of CAP-based fluoroscopy studies should be acknowledged. In a study assessing CAP myelography and CAP-CT myelography as advanced imaging modalities for IDDS treatment failure, Delhaas et al. reviewed 70 CAP procedures, of which 24% could not be aspirated. CAP myelography demonstrated limited diagnostic performance, with a sensitivity of 52% and specificity of 86% compared with reference tests that were determined on a case-by-case basis using available imaging, clinical, and surgical data. A key limitation of this study was the absence of a consistent reference standard and the retrospective nature of image review [29].

Fluoroscopy alone may yield false-negative results due to the limited ability to detect catheter-tip loculations, subtle catheter migrations, or microscopic perforations and leaks [33]. In a small study of seven patients with suspected intrathecal baclofen system malfunction, Shapiro and colleagues evaluated combined C-arm fluoroscopy and C-arm cone beam computed tomography (CCBT). While the combined approach was effective in identifying problems and guiding intervention, fluoroscopy alone was associated with poor visualization, inability to confirm normal function, uncertainty regarding pump-catheter disconnection, and visualization of contrast leakage without clear localization of the defect [36].

### 6.2 Computed tomography including catheter access port CT-myelography

Given the diagnostic limitations of plain radiographs and fluoroscopy evaluation, CT-based imaging may be considered as the next step in the diagnostic algorithm. After radiographs suggest an intact catheter and CAP aspiration confirms cerebrospinal fluid return, CT myelography can be performed to assess flow of contrast through the catheter into the subarachnoid space. CT myelography enables evaluation of catheter patency, leakage, dislodgement, and improper catheter-tip location while providing high-resolution visualization of the surrounding anatomy [29].

In their troubleshooting algorithm, Saulino et al. recommend catheter imaging beginning with plain radiographs, followed (if catheter patency is confirmed by successful CAP aspiration) by injection of contrast through the CAP with visualization using either fluoroscopy or CT. They emphasize that CT myelography offers the added advantage of providing structural information about adjacent anatomy that may represent potential noxious stimuli. To fully visualize the pump and entire catheter course, CT imaging should encompass the abdomen as well as the thoracic and lumbar spine [6].

The added diagnostic value of CT is highlighted in cases where fluoroscopy is inconclusive. Ellis et al. described the use of high-resolution 3D-CT to evaluate an IDDS after two fluoroscopic contrast studies failed to identify a block or leak. Following the second nondiagnostic fluoroscopic study, a high-resolution noncontrast lumbar spine CT with 0.6 mm slice thickness revealed a faint blush of contrast in the posterior paraspinal soft tissue around the catheter. 3D reconstruction confirmed extravasation of the contrast, prompting surgical replacement. Gross inspection of the catheter revealed no visible defect, suggesting a possible microfracture as the underlying cause of malfunction [37].

Several studies further support the utility of CT-based evaluation. In a study investigating the use of CT imaging to assess intrathecal baclofen delivery system dysfunction, 27 patients with SynchroMed infusion systems underwent CT following standard preliminary evaluation, including exclusion of clinical change, pump interrogation, and plain radiography. The protocol involved aspiration of the CAP for CSF followed by injection of 1-2 ml of contrast through the access port; if CAP aspiration was unsuccessful, noncontrast CT with three-dimensional reconstruction was performed.

Of the 27 patients, CAP aspiration was unsuccessful in three cases. Eight patients had normal CT findings, while 16 had abnormalities that prompted revision surgery. Five cases showed inadequate contrast pooling, suggesting partial occlusion of the catheter from arachnoid tissue or subdural migration. Five additional patients had fluid leak identified on CT; intraoperative findings revealed two pump-catheter connector leaks, one pinhole catheter defect that was managed with trimming and reconnection, and two cases requiring complete catheter replacement. Two patients were found to have catheter occlusion, visualized on CT as confined fluid loculation at the catheter tip without evidence of free intrathecal fluid flow [38]. The specific pump generation was not reported. Although the study period spanned 2011–2015, it included both newly implanted systems and patients already receiving intrathecal therapy, limiting definitive attribution to a particular SynchroMed model.

Advanced CT reconstruction techniques may further enhance diagnostic yield. In a study comparing contrast enhanced three-dimensional CT with volume rendering technique (VRT) to fluoroscopy and axial CT, complications related to 60 pumps implanted for either baclofen or morphine delivery were reviewed. After conventional radiographs were nondiagnostic in 35 cases of possible malfunction, high-resolution 3D CT VRT was obtained. Seven catheter-related abnormalities were identified, prompting surgical revision, while 28 scans were unremarkable and surgery was avoided. Among 12 cases who underwent fluoroscopy, four cases underwent revision surgery, with fluoroscopy identifying the underlying cause in only half of cases. High-resolution 3D CT with VRT provided superior visualization of the entire catheter course, including the pump connection, area posterior to the pump, spinal entry point, and intrathecal trajectory, compared with axial CT alone [39].

In situations where CAP aspiration is not feasible or nuclear medicine studies are unavailable, lumbar puncture with intrathecal contrast may be considered. Subsequent imaging, often CT myelography, allows for assessment of CSF fluid dynamics and tracer distribution along the spinal canal and catheter tip. Delhaas et al. described 17 cases in which CSF could not be aspirated from the CAP; in one patient, lumbar puncture CT myelography was performed to confirm epidural catheter position [29].

### 6.3 Nuclear medicine scintigraphy

**Fig. 7:**
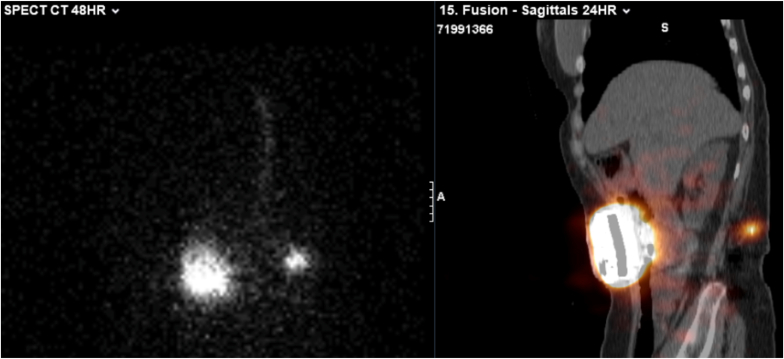
SPECT/CT Localization of Intrathecal Catheter Leak Planar gamma camera images and SPECT/CT of the lower abdomen and pelvis were obtained at 24 and 48 h following In-111 DTPA injection into the intrathecal pump reservoir. SPECT/CT demonstrates focal radiotracer activity at the L3 level, corresponding to the catheter transition from subcutaneous tissue through the musculature before entering the spinal canal, consistent with a catheter leak. Mild activity is also visualized within the intrathecal portion of the catheter.

If CAP fluid aspiration is not possible or if CT myelogram is nondiagnostic, nuclear medicine scintigraphy may be considered as a subsequent imaging modality. Indium-111 diethylenetriamine pentaacetic acid (DTPA) single-photon emission computed tomography combined with CT (SPECT-CT) allows for assessment of dynamic intrathecal flow and pump function over time (Fig. 7). This modality can identify subtle leaks or micro-disconnections, including tracer leakage posterior to the pump, restriction of activity to the pump reservoir, interruption of activity along the catheter course, abnormal tracer pooling suggestive of a subcutaneous pocket, and failure of tracer migration to the intended intrathecal destination [[40], [41], [42]].

The diagnostic utility of radionuclide studies has been demonstrated in patients with otherwise unrevealing conventional imaging. Yowtak et al. evaluated 23 patients with suspected IDDS malfunction and normal radiographic imaging and identified pump malfunction in 19 patients using radionuclide studies. One of the main advantages of this imaging modality is the ability to assess pump function under normal operating conditions. Because the injected volume of radionuclide agent is small, the tracer can be introduced into the pump reservoir using a standard refill technique without requiring catheter aspiration, significantly altering drug concentration, or reprogramming the pump [41].

### 6.4 Magnetic resonance imaging

MRI of the spine may be obtained with and without gadolinium administration when there is a high clinical suspicion for concomitant structural pathology, such as spinal cord compression, mass lesions (e.g, catheter-tip granulomas) or arachnoiditis. MRI provided superior soft-tissue contrast and may help identify extrinsic or intrinsic spinal pathology that is otherwise not well characterized by fluoroscopic, CT-based, or nuclear medicine imaging.

However, MRI has important limitations in evaluating catheter position and integrity. Dardashti et al. described a case in which MRI suggested intramedullary migration of an intrathecal catheter tip. Following laminectomy, the catheter was determined to be surrounded by subarachnoid adhesions as opposed to intramedullary. This case highlights the potential for MRI to mischaracterize catheter location, as differentiation between the spinal cord and adjacent adhesions may be unreliable [43].

## 7 Decision-making algorithm

### 7.1 Step 1: triage and stabilization

Evaluation should begin by identifying scenarios in which treatment must precede diagnostic testing. Presentations concerning for intrathecal drug overdose, acute withdrawal, new or progressive neurologic deficit, suspected catheter-tip inflammatory mass, or severe CSF leak or infection warrant immediate supportive management while troubleshooting continues. Device failures and human-factor errors (e.g., refills or programming) can precipitate life-threatening overdose or withdrawal syndromes. In high-risk or rapidly evolving presentations, early escalation to an experienced center and multi-disciplinary involvement are appropriate, even as diagnostic evaluation proceeds in parallel.

### 7.2 Step 2: focused bedside assessment and pump interrogation

**Fig. 8:**
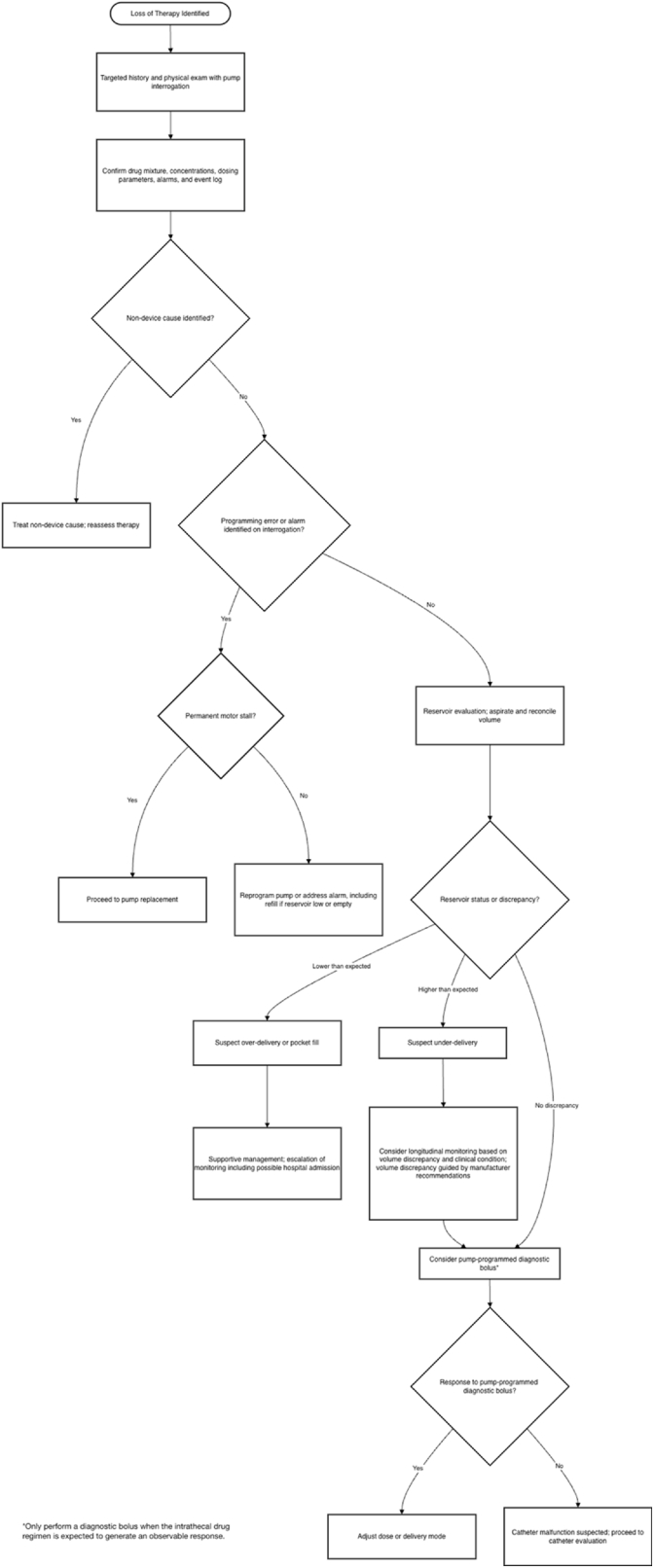
**Loss of therapy algorithm.** Approach to evaluating diminished or absent therapeutic effect in patients with intrathecal drug delivery systems.

**Fig. 9:**
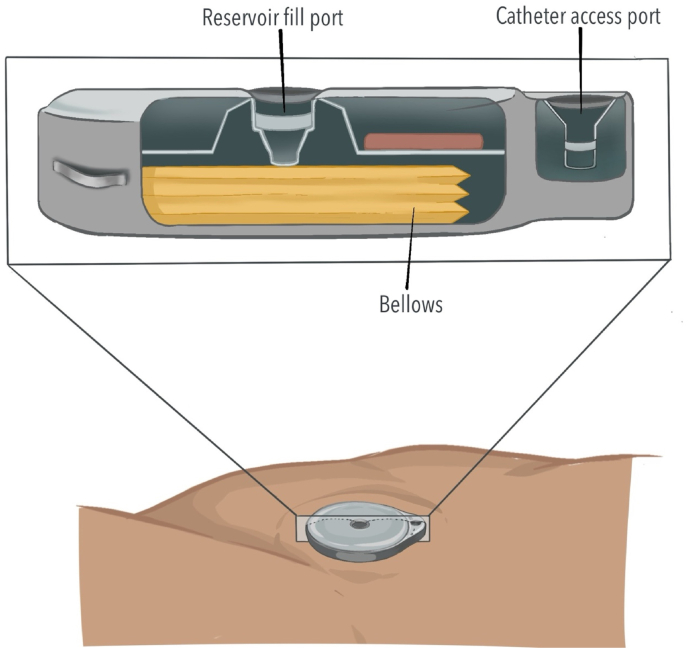
Typical positioning of an intrathecal drug delivery system Subcutaneous placement of the pump, most commonly in the lower abdominal quadrant.

Once the patient is stabilized, a focused bedside assessment should determine whether the clinical presentation is more consistent with true drug delivery failure versus non-mechanical loss of efficacy (Fig. 8). This includes correlating symptom onset and trajectory with recent pump refills, programming changes, alarms, or external environmental factors (e.g., MRI exposure). Formal pump interrogation is also necessary to evaluate battery life, end-of-service indicators, error logs, and programmed dosing parameters. This stage helps distinguish pump-level malfunction from catheter-related or pharmacologic causes (Fig. 9).

### 7.3 Step 3: reservoir volume reconciliation

Reservoir volume reconciliation is the initial functional evaluation of system integrity. The aspirated reservoir volume should be compared with the expected volume calculated from pump interrogation. Excess residual volume raises concern for underdelivery due to catheter obstruction or pump malfunction, whereas lower-than-expected volume suggests leakage, pocket fill, or overinfusion. These findings inform whether catheter-level evaluation is necessary and guide downstream imaging decisions.

### 7.4 Step 4: initial decision point

If reservoir volume is appropriate, pump interrogation does not suggest pump malfunction, and the clinical presentation does not strongly suggest device-related malfunction, clinicians should consider other contributors to the clinical presentation, including pharmacologic tolerance, disease progression, catheter-tip granuloma, opioid-induced hyperalgesia, or psychosocial factors. In this scenario, further device work-up may be deferred in favor of regimen optimization and adjunctive therapies and close clinical follow-up. If diagnostic uncertainty persists or the presentation remains concerning for mechanical failure, catheter-level evaluation is indicated.

### 7.5 Step 5: catheter access port aspiration

**Fig. 10:**
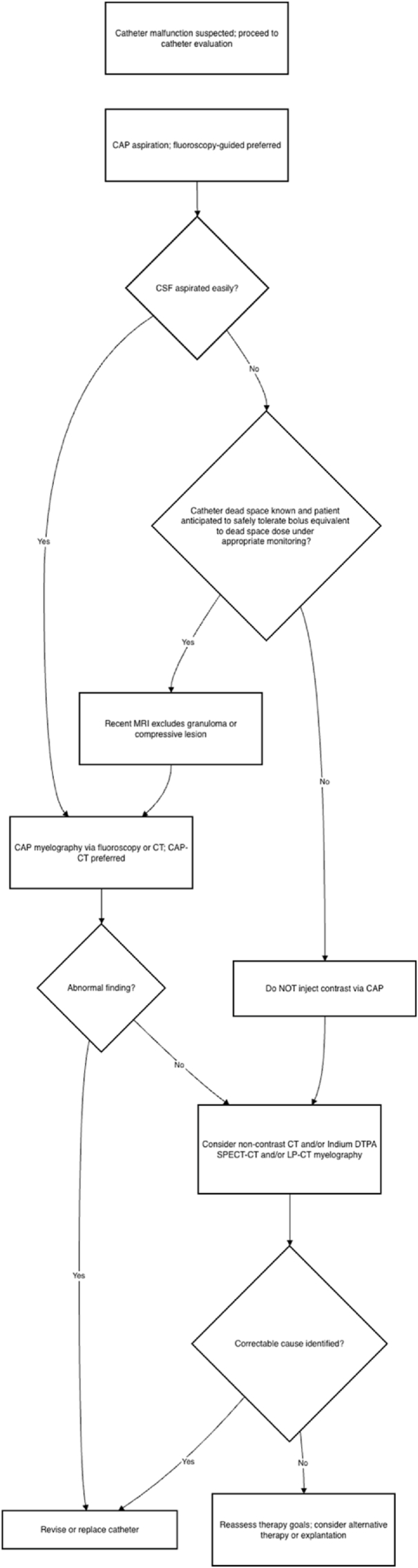
Catheter evaluation algorithm. Approach to suspected intrathecal catheter dysfunction. CAP = catheter aspiration port, CSF = cerebral spinal fluid, MRI = magnetic resonance imaging, CT = computed tomography, DTPA = diethylenetriamine pentaacetic acid, SPECT = single-photon emission computed tomography, LP = lumbar puncture.

Catheter access port aspiration is the initial step in catheter-level evaluation after the pump assessment and reservoir reconciliation suggest the need for further investigation (Fig. 10). Aspiration under strict sterile technique serves two purposes: confirming catheter patency and establishing whether contrast-based studies can be safely performed. Successful aspiration of CSF supports catheter continuity and permits contrast injection through the CAP. Failure to aspirate does not reliably confirm obstruction or disconnection.

Before any catheter-level contrast bolus is considered, recent MRI imaging should have excluded catheter-tip granuloma or other compressive lesions. In the absence of such contraindications, contrast injection through the CAP may still be considered if the catheter dead space has been accurately calculated and appropriate contingencies are in place. This includes explicit knowledge of the volume and concentration of medication within the catheter, estimation of the potential intrathecal bolus that may be delivered with contrast injection, assessment of the patient's clinical tolerance to such a dose, and preparation for monitoring and supportive management should overdose or adverse effects occur. If these conditions cannot be met, contrast injection should be deferred, and evaluation should proceed with imaging modalities that do not require catheter injection.

### 7.6 Step 6a: advanced imaging - CAP aspiration successful

If CAP aspiration is successful, advanced imaging with intrathecal contrast can be performed. Although fluoroscopic CAP myelography may be used as an initial screening study, its diagnostic sensitivity is limited. Given this false-negative rate, clinicians may reasonably proceed directly to CAP-CT myelography with high-resolution 2D and 3D reconstructions, particularly when clinical suspicion for mechanical failure remains high.

CAP-CT myelography should encompass the entire catheter course, including the intrathecal segment, extravertebral tract, pump-catheter connector, and abdominal pocket. This approach improves detection of subtle defects, leakage, migration, or malposition that may not be apparent on fluoroscopy alone. Contrast identified near the pump pocket should be interpreted carefully, as it may represent retrograde tracking from a dural leak as opposed to a primary pocket fill.

### 7.7 Step 6b: advanced imaging - CAP aspiration unsuccessful

If CAP aspiration is unsuccessful and contrast injection through the catheter cannot be safely pursued, despite knowledge of catheter dead space, drug concentration, and appropriate contingency planning, diagnostic evaluation should proceed with modalities that do not require catheter-based contrast injection. Indium-111 DTPA scintigraphy with SPECT-CT can assess dynamic flow and tracer distribution under physiologic pump operating conditions without altering intrathecal drug delivery. Lumbar puncture CT myelography may also be considered to evaluate catheter position, contrast spread, or anatomic obstructions when prior studies are inconclusive. MRI should be reserved for evaluation of suspected catheter-tip granuloma, syrinx, arachnoiditis, or other compressive spinal pathology.

### 7.8 Step 7: management

Management should be driven by the results of functional assessment and imaging, with several overarching principles helping to inform next steps. Expected reservoir volume, preserved catheter integrity, and unremarkable imaging support medical optimization rather than surgical intervention. Unexpected reservoir discrepancies with preserved catheter patency suggest pump-level failure and warrant planning for pump revision or replacement. Localized catheter defects, leaks, disconnections, or malposition should prompt targeted surgical revision. Identification of a catheter-tip inflammatory mass or compressive lesion mandates immediate cessation of intrathecal drug delivery and urgent neurosurgical consultation, particularly in the presence of neurologic deficits.

Building on these principles, we now present two practical, step-by-step algorithms, one for suspected intrathecal overdose or withdrawal and one for loss of therapeutic effect. These protocols reflect our synthesis of the primary literature combined with clinical experience. They are intended as pragmatic frameworks and should be contextualized to local resources.

### 7.9 Step 7a: overdose and withdrawal

**Fig. 11:**
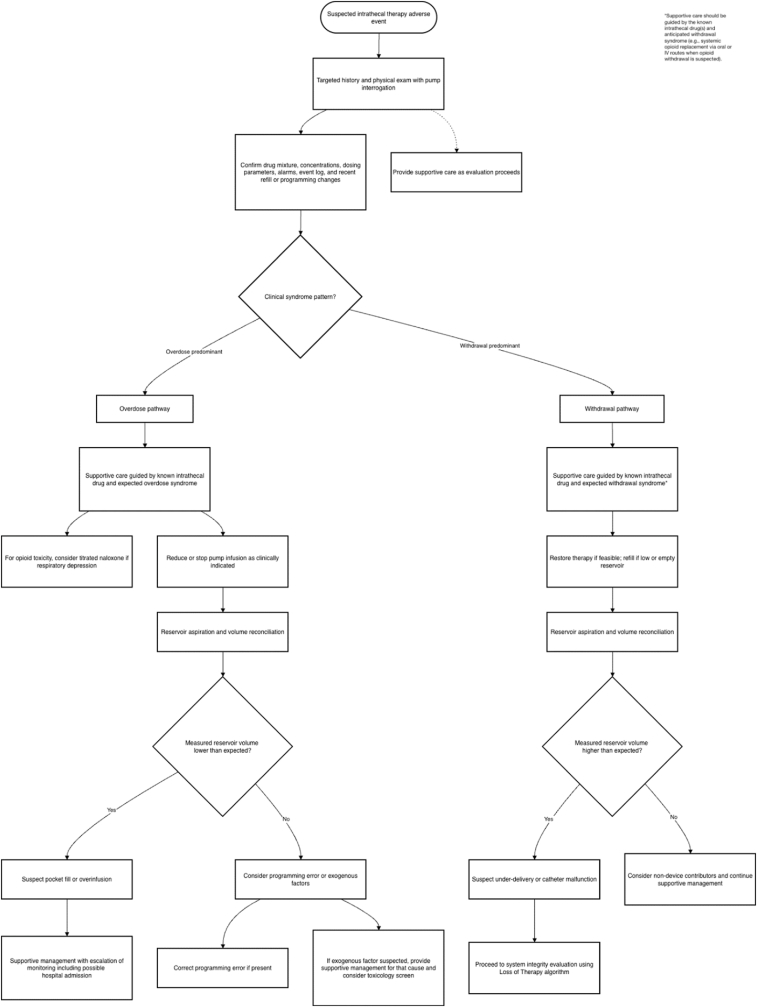
Overdose and withdrawal algorithm. Approach to suspected intrathecal drug delivery system overdose or withdrawal.

If there is concern for overdose or withdrawal in a patient with an IDDS, the first step is to perform a targeted history and physical exam along pump interrogation (Fig. 11). Pump interrogation is performed to confirm the drug mixture, determine whether there have been any changes to dosing and delivery parameters, and review alarms, event logs and recent refills. Supportive care should be provided concurrently with the initial evaluation. Two general clinical syndromic patterns may be observed: overdose-predominant and withdrawal-predominant. If overdose is suspected, supportive care should be guided by the intrathecal drug being administered, as overdose presentations vary by medications. For example, in cases of suspected opioid toxicity, titrated naloxone should be utilized to mitigate opioid-induced respiratory depression. If overinfusion is suspected, consideration should be given to reducing the infusion rate or stopping the pump.

The next step is reservoir aspiration to help elucidate the underlying cause. If the reservoir volume is lower than expected, overinfusion or pocket fill should be suspected. Supportive care should be escalated, and admission to the hospital for monitoring should be considered. If there is no discrepancy between the expected and actual reservoir volume, programming error or an exogenous source of overdose should be considered. A toxicology screen can help identify potential exogenous contributors.

If withdrawal is suspected, the initial step is to provide supportive care tailored to the intrathecal drug and the expected withdrawal syndrome for that drug. For example, if the intrathecal agent is an opioid, systemic administration via intravenous or oral routes is required. Similarly, if clonidine withdrawal is suspected, intravenous administration of clonidine and management of sympathetic symptoms such as hypertension are necessary. It should be acknowledged that multiple agents may be present in the pump, resulting in a mixed withdrawal pattern. Next, therapy should be restored if feasible. If the reservoir is low or empty, it should be refilled to restore therapy. If restoration is not possible, reservoir aspiration and volume reconciliation should be performed. If the true reservoir volume is equal to or less than expected, non-device contributors should be considered, and supportive care continued. If the true reservoir volume is higher than expected, under-delivery or catheter malfunction should be suspected and a system integrity evaluation using the Loss of Therapy algorithm should be conducted.

### 7.10 Step 7b: loss of therapy

If loss of therapy is suspected, the first step is to perform a targeted history and physical exam along with pump interrogation. The drug mixture, concentration and dosing parameters should be confirmed, and any alarms or entries in the event log reviewed. If a non-device cause is identified, it should be treated, and therapy reassessed afterward. If a programming error or alarm is identified on device interrogation, the device should be assessed for motor stall. If permanent motor stall is detected by the system, the pump should be surgically replaced. If no motor stall is identified, the pump should be reprogrammed or alarms addressed. This includes refilling the reservoir if it is low or empty. If pump interrogation does not reveal a cause, reservoir aspiration and volume reconciliation should be performed. If the reservoir volume is less than expected, over-delivery or pocket fill should be suspected. In this case, supportive care should be provided and escalated as needed, as the patient may require admission to the hospital for monitoring. If the reservoir volume is higher than expected, drug under-delivery should be suspected. In cases of suspected under-delivery, consideration of longitudinal monitoring may be appropriate based on the volume discrepancy and patient's clinical condition. Management of specific magnitudes of volume discrepancy can also be guided by manufacturer recommendations.

A pump-programmed diagnostic bolus may be considered when there is a higher-than-expected volume or no volume discrepancy. If the patient has an appropriate response to the bolus, dose adjustment or changes to the delivery mode should be considered. A diagnostic bolus should only be considered if an expected noticeable response is anticipated based on the agents in the pump. If there is no response to the pump-programmed bolus, catheter malfunction should be suspected, and the clinician should proceed with catheter evaluation. Fluoroscopy-guided CAP aspiration is recommended.

If CSF is aspirated easily through the CAP, the catheter is assumed to be patent, and it is appropriate to proceed with CAP myelography via fluoroscopy or CT. In these cases, the entire catheter volume can be aspirated with CSF, and injection of contrast will not bolus any drug through the catheter. CAP-CT is the preferred method of assessment. If this reveals an abnormal finding, the catheter should be revised or replaced. If there is no abnormality on CAP myelography, the clinician may consider non-contrast CT, nuclear medicine scintigraphy or lumbar puncture CT myelography. If a correctable cause is identified on further imaging, the catheter should be revised or replaced. If a cause cannot be identified, therapy goals should be reassessed, alternative therapy options explored, and pump explantation considered.

If CSF cannot be aspirated easily, the volume of catheter dead space should be calculated. If the volume of dead space is known or can be determined, the patient is anticipated to safely tolerate a bolus, and adequate monitoring is available, a contrast bolus injection may be considered. This presumes that injection of contrast through the catheter has the potential to bolus intrathecal drug if catheter volume cannot be cleared by aspirating CSF. Prior to proceeding with injection, recent MRI should be reviewed to confirm the absence of granuloma or compressive lesion. If there are no contraindications, CAP myelography with fluoroscopy or CT is appropriate. If an abnormal finding is identified, revision or replacement of the catheter is recommended. If no abnormalities are identified via CAP myelography, alternative imaging should be considered (non-contrast CT, indium DTPA SPECT-CT and/or LP-CT myelography).

If the dead space is unknown, the patient is not anticipated to safely tolerate a bolus, appropriate monitoring is not available, and/or there is concern for a granuloma, contrast injection via the CAP is not recommended. Alternative imaging such as non-contrast CT and/or Indium DPTA SPECT-CT and/or LP-CT myelography should be considered. If a correctable cause is identified on imaging, catheter revision or replacement is recommended. If a cause is unable to be identified, therapy goals should be reassessed, pump explantation and alternative therapies should be considered.

## 8 Conclusion

Troubleshooting IDDS requires an organized approach to guide efficient care. The prompt and accurate identification of IDDS malfunction is crucial to preventing morbidity, including withdrawal syndromes, overdose, and neurological injury. A stepwise framework, from clinical evaluation to imaging to functional assessments, encourages efficiency while minimizing unnecessary interventions. When basic assessments are inconclusive, early use of advanced imaging can help localize subtle failures. Clinicians must be familiar with device-specific behaviors and MRI safety protocols to prevent IDDS dysfunction. The effective management of IDDS involves interdisciplinary collaboration, with neurosurgery, radiology, and device manufacturers, to guide system failures and restore therapeutic effect. Ultimately, the safe and effective use of IDDS requires clinical vigilance, technical knowledge, and thoughtful diagnostic reasoning.

## Declaration of interest

We have no conflicts of interest to report.
